# Mechanical Experiments on Concrete with Hybrid Fiber Reinforcement for Structural Rehabilitation

**DOI:** 10.3390/ma15082828

**Published:** 2022-04-12

**Authors:** Muhammad Asharib Shahid, Muhammad Usman Rashid, Nazam Ali, Krisada Chaiyasarn, Panuwat Joyklad, Qudeer Hussain

**Affiliations:** 1 Civil Engineering Department, University of Management and Technology, Lahore 54770, Pakistan; asharibshahid@gmail.com (M.A.S.); usman.rashid@umt.edu.pk (M.U.R.); nazam.ali@umt.edu.pk (N.A.); 2 Thammasat Research Unit in Infrastructure Inspection and Monitoring, Repair and Strengthening (IIMRS), Thammasat School of Engineering, Faculty of Engineering, Thammasat University Rangsit, Pathum Thani 12000, Thailand; ckrisada@engr.tu.ac.th; 3 Department of Civil and Environmental Engineering, Faculty of Engineering, Srinakharinwirot University, Nakhonnayok 26120, Thailand; 4 Center of Excellence in Earthquake Engineering and Vibration, Department of Civil Engineering, Chulalongkorn University, Bangkok 10330, Thailand; ebbadat@hotmail.com

**Keywords:** rehabilitation, Kevlar fibers (KF), glass fibers (GF), high-performance fiber-reinforced concrete (HPFRC)

## Abstract

Reinforced concrete is used in the construction of bridges, buildings, retaining walls, roads, and other engineered structures. Due to seismic activities, a lot of structures develop seismic cracks. The rehabilitation of such structures is necessary for public safety. The overall aim of this research study was to produce a high-performance hybrid fiber-reinforced concrete (HPHFRC) with enhanced properties as compared to plain high-performance concrete and high-performance fiber-reinforced concrete (HPFRC) for the rehabilitation of bridges and buildings. Kevlar fibers (KF) and glass fibers (GF) with lengths of 35 mm and 25 mm, respectively, were added and hybridized to 1.5% by mass of cement to create hybrid fiber-reinforced concrete mixes. Eight mixes were cast in total. The compressive strength (*f*′_c_), flexural strength (*f*_r_), splitting tensile strength (*f*_s_), and other mechanical properties, i.e., energy absorption and toughness index values, were enhanced in HPHFRC as compared to CM and HPFRC. It was found that the concrete hybridized with 0.75% KF and 0.75% GF (HF-G 0.75 K 0.75) had the most enhanced overall mechanical properties, illustrating its potential to be utilized in the rehabilitation of bridges and structures.

## 1. Introduction

Concrete is the most frequently used material in structures around the globe. Concrete deteriorates over time, while seismic activities can also reduce the life of a structure by damaging it. Except for a few conventional materials, other advanced materials are not yet well developed or universally implemented and their repair capabilities and long-term maintenance requirements have not been investigated [1]. Structures built in the 1960s and 1970s, which are now 50–60 years old, require rehabilitation or else they will pose a hazard to public safety. Among the various methods of concrete rehabilitation, the use of fiber-reinforced concrete (FRC) for rehabilitation and retrofitting has become very popular in the last decade, and significant studies have been performed on FRC [2,3,4]. As is clear from its name, FRC contains randomly distributed fibers in the concrete mix in all dimensions, which improves the tensile properties, help in resisting cracks, and make the concrete more ductile [5]. Several types of fiber, including natural and synthetic fibers, have been added to concrete, which in turn enhanced the performance [6]. To achieve the required properties, which cannot be achieved with the use of one fiber type in FRC, researchers have used more than one fiber type to utilize the synergetic effects of both in order to obtain the required mechanical properties, known as hybrid fiber-reinforced concrete (HFRC). Fiber hybridization is the procedure of combining and maximizing the advantages of fibers in an effective way [7]. The two main methods of fiber hybridization are hybridization according to the fibers’ mechanical properties and hybridization according to the size of fibers. In this study, both methods are incorporated to enhance the results. The compressive strength, flexural strength, splitting tensile strength, and corresponding energy absorption and toughness index values are measured using ASTM testing methods. Damaged structures can no longer absorb the energy produced due to dynamic loading, so rehabilitation materials must be tough and show good energy absorption to dissipate the energy produced; as such, energy absorption and toughness are given prime importance in this research. Table 1 shows the literature on hybrid fiber-reinforced concrete in the last two decades, along with the different fiber combinations and major conclusions.

In recent years, a lot of high-strength fibers have been made available on the market, which can be used to make even more durable and long-lasting concrete for rehabilitation purposes. The concrete itself must have high performance to incorporate these fibers and in order to use their strength to the full extent. High-performance FRC and high-strength FRC are growing rapidly due to the design requirements for reducing the mass of structures and smaller sections, which ultimately has led to the cost-effective design of seismic-resistant structures. Fibers in ultra-high-performance concrete show improved properties and have been proven as excellent overlay rehabilitation materials with excellent bond strength when combined with previously damaged concrete [8]. Each fiber type has its own specific characteristics, which when added in concrete form an enhanced composite. Fibers are selected according to their different properties, such as their diameter, specific volume, tensile strength, and Young’s modulus. Steel wool microfibers in ultra-high-performance concrete provide the best properties for the rehabilitation and widening of bridges [9]. The fibers in the concrete prevent it from cracking, which is the major cause of structural vulnerability to damage [10].

Many researchers have worked on the rehabilitation of structures using different materials. Normally damaged concrete components are rehabilitated by overlaying rehabilitation materials. Jacketing columns can increase the load-carrying capacity and can also help gain lost strength; the load-carrying capacity increases with increases in the jacketing area [11]. Another method involves circularizing columns, which provides significant increases in the axial load-carrying capacity, ultimate flexural load-carrying capacity, and energy absorption capacity [12]. Beams can also be rehabilitated through jacketing with reinforcements and bolts. U-shaped precast jackets fixed with lateral bolts and overlay jackets have been proven to increase the ultimate load-carrying capacity after rehabilitation [13]. For the rehabilitation of bridge decks and slabs, an additional layer of ultra-high-performance fiber-reinforced concrete (UHPFRC) is added on the top for protection, and a layer of reinforced ultra-high-performance fiber-reinforced concrete (R-UHPFRC) is added for structural resistance. This method has been adopted in the field and has been proven to provide significant improvements after rehabilitation [14]. The jacketing of damaged foundations is also an effective method of rehabilitation.

**Table 1:** Some of the available studies on hybrid fiber-reinforced concrete.

Reference	Limitations of Fiber Contribution	Major Conclusions
Chen et al., 2020 [15]	PF (0.03–0.09%), SF (0.5–1%) by volume	UHPC with 0.03% PF and 0.5% steel fibers showed best results at temperatures of 300 °C, 400 °C, and 500 °C in compression and flexural strength but splitting tensile strength reduced. PF fibers burn and help reduce internal water pressure.
Smarzewski, 2019 [16]	SF (0.5%, 1%), PF (0.06%) by volume	The hybridization of PF and SF in HFC prevented cracks, improved the peak strength, and increased the energy absorption and ductility index.
Al-Gemeel et al., 2018 [17]	HGM (0% and 10%), PVA (0.12–1.75%), SF (0.25–0.75%) by volume	Compressive and flexural strengths of HFM mix were noted to decrease. Overall properties enhanced via hybrid fiber energy absorption, which had little effect due to the use of HGM
Khan et al., 2018 [18]	CaCO_3_ whiskers, SF (0.9%), BF (0.34–1.36%) by volume	Increases were observed in compressive, flexural, and splitting. tensile strengths. The best results were exhibited by concrete with SF (0.32%, CaCO_3_ (0.9%), and BF 0.68%.
Dawood and Ramli, 2018 [19]	SF (0.25–2%), palm fiber (0.25–1%), barchip fiber (0.25%, 0.5%) by volume	Modulus of elasticity increased by 25–34% in the best hybridized mix. Decreased permeability and increased compressive strength observed.
Afroughsabet and Ozbakkaloglu, 2015 [20]	SF (0.25–1%), PF (0.15–0.45%) by volume	Mechanical properties of HSC were improved by hybrid fibers. Here, 0.85% SF and 0.15% PF exhibited the best results.
Almusallam et al., 2015 [21]	(0.7–1.4%) SF, (0.2%) PF, (0.3%) KF by volume	A hybrid fiber-reinforced slab’s impact resistance was affected more by geometrical rather than material properties.
Chi et al., 2014 [22]	(0.5–1.5%) SF, (0.05–0.15%) PF by volume	Compressive strength increases with volume increase in PF. HFRC exhibited better performance post-peak.
Soe et al., 2013 [23]	SF (0.5–0.58%), PVA (1.5–1.75%) by volume	Mix with 1.75% PVA fibers and 0.58% steel fibers exhibited improvements.
Bajaj et al., 2012 [24]	SF (0·125–1·125%), PF (0·125–1·125%) by volume	Hybrid fiber-reinforced mix with equal percentages of both fibers exhibited the best flexural fatigue results.
Dawood and Ramli, 2011 [25]	SF (1–2%). Palm fiber (0.25–1%) by volume	The addition of 1% pf steel fibers increased the strength by 13%. The hybrid combination of 1.5% SF + 0.5% palm fiber significantly increase the toughness index.
Banthia and Sappakittipakorn 2007 [26]	SF of different diameters (0.25–0.75%) by volume	Toughness enhanced when large-diameter fibers replaced by small-diameter fibers.
Sivakumar and Santhanam, 2007 [27]	(0.12–0.5%) SF, (0.12–0.5%) PF, (0.12–0.5%) GF by volume	Flexural strength and toughness enhanced by hybrid fibers.
Ahmed et al., 2007 [28]	(0.5–2.5%) SF, (1–2.5%) PEF, (1–2.0%) PVA by volume	The steel–PVA hybrid exhibited higher flexural strength, but lower deflection as compared to steel–PE. The post-cracking strength of steel–PE was greater as compared to steel–PVA
Hua et al., 2005 [29]	CF, PF, GF, PE	Compressive strength was not affected by length/diameter ratio, but flexural strength was impacted.
Lawler et al., 2005 [30]	(0.32% macro and micro) SF, (0.5%) PVA by volume	Micro-SF mix was found to resist macro-cracks by delaying them as compared to the mix with macro-SF only.
Yao et al., 2003 [31]	(0.2–0.5%) SF, (0.3–0.5%) PF, (0.2–0.5%) CF	High strength and flexural toughness were noted for carbon–steel hybrid combination because of the synergetic effects of their similar properties.
Lawler et al., 2002 [32]	SF, PF	Reduction in permeability of hybrid fiber-reinforced mortar post-cracking was observed due to hybridization.
Ramanalinagm et al., 2001 [33]	PVA, SF (micro and macro)	Peak load and post-peak ductility enhanced by hybrid fiber reinforcement.
Sun et al., 2001 [34]	(0.25–1.5%) SF, (0.25–1.5%) PF, (0.25–1.5%) PVA by volume	By improving the pore structure of the concrete, hybrid fibers hindered crack formation.

**Notes**: CF, carbon fiber; GF, glass fiber; KF, Kevlar; PEF, polyethylene fibers; PF, polypropylene fibers; PVA, polyvinyl alcohol; SF, steel fibers; BF, basalt fibers; HGM, hollow glass microspheres. **Note**: The same fibers did not necessarily have the same geometrical and mechanical properties when used in different studies, meaning the results may have varied.

## 2. Research Motivation and Significance

The research motivation is to provide an enhanced material for the rehabilitation of structures, which is emerging as a large industry around the globe, as concrete requires repair after exposure to harsh environmental effects, wear and tear, and seismic activities. If rehabilitation is not properly performed, the structure at risk may fail, which can result in many causalities. Cracks that develop in structures due to seismic activity may cause moisture to reach the reinforcement and cause corrosion [35]. Corrosion of reinforcements is the leading cause of structural damage and premature degradation of RCC structures [36]. The research significance of high-performance concrete with hybrid fiber reinforcement using Kevlar and glass fibers for the rehabilitation of damaged bridges and structures is yet to be investigated. The motive of this research is to incorporate the synergetic effects of hybrid fibers into high-performance concrete to obtain a better rehabilitation material in order to improve infrastructure and increase public safety by eliminating the hazards posed by damaged structures. The innovative aspect of this research study is the creation of a high-performance hybrid fiber-reinforced concrete (HPHFRC) with enhanced properties as compared to plain high-performance concrete and high-performance fiber-reinforced concrete (HPFRC) for the rehabilitation of bridges and structures.

## 3. Methodology

**Figure 1:**
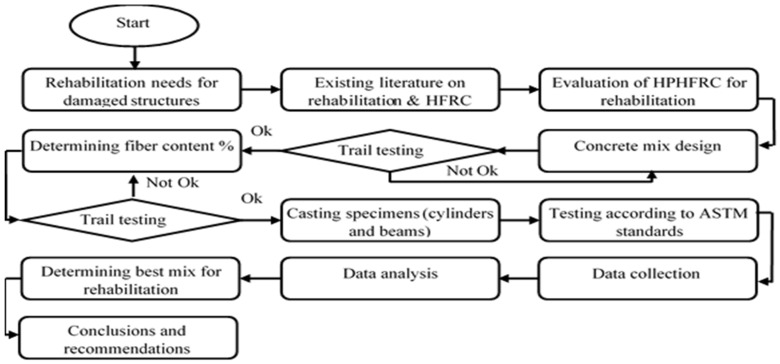
Methodology flow chart of the research work.

For the rehabilitation of concrete bridges and structures, Kevlar and glass fibers were hybridized in high-performance concrete in different percentages to evaluate their behavior and mechanical properties, keeping in mind the previous literature on hybrid fiber-reinforced concretes and rehabilitation. The mix designs and fiber percentages were chosen on the basis of the most suitable results in the trial testing. The materials were physically investigated before the mixes were prepared. ACI 211 guidelines were used to prepare the mix designs. The flow chart of this research study is shown in Figure 1. In the first step, the existing literature was evaluated to assess the need to rehabilitate damaged structures using HPHFRC. In the next steps, the concrete mixes were designed, and tests were performed according to ASTM standards. Finally, the best concrete mixes were recommended based on data collection and analysis for the purpose of rehabilitating concrete bridges.

## 4. Experimental Program

### 4.1. High-Performance Concrete

**Figure 2:**
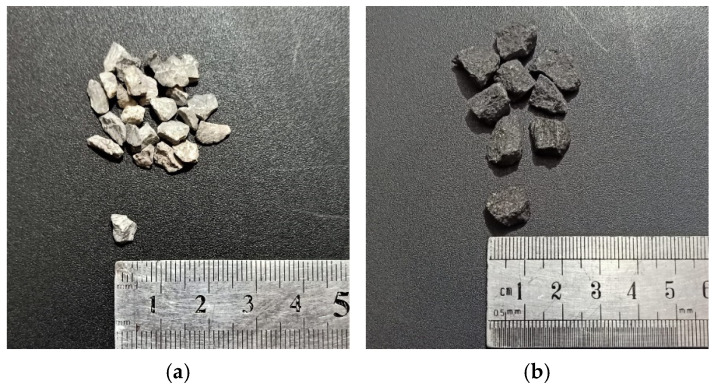
(**a**) Margala crush 5 mm and (**b**) granite crush measuring 10 mm.

The ingredients were batched by weight at a mix design ratio of 1:1.2:1.8 (cement/sand/aggregate) with 8% silica fumes (SF) and 0.6% high-performance water-reducing agent by mass of cement, mixed at a W/C + SF ratio of 0.31 for the high-performance concrete named the control mix (CM). The mix design ratio was selected after trial testing. The mix proportion for the control mix is shown in Table 2. Two types of aggregates were used, namely granite and Margala crush (10 and 5 mm), as shown in Figure 2.

### 4.2. Fibers

**Figure 3:**
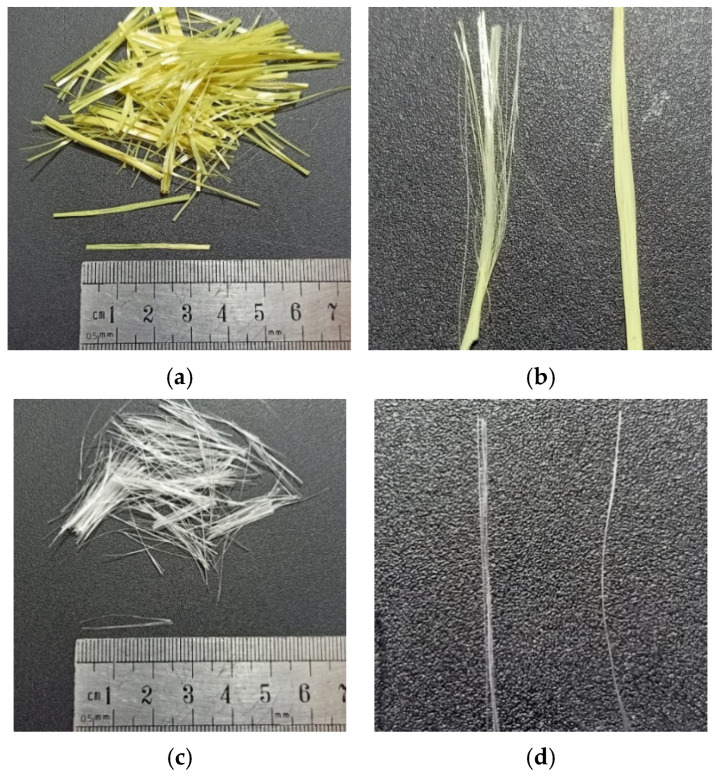
Fibers used in the study: (**a**) Kevlar fibers; (**b**) Kevlar bundles; (**c**) glass fibers; (**d**) glass bundles.

Kevlar and glass fibers were used in this study. The type of Kevlar used was Kevlar 29 and type of glass used was E-Glass fibers, as shown in Figure 3. The physical and mechanical properties of both fibers are shown in Table 3. The fibers used were bundled, which upon mixing in wet concrete dispersed homogeneously.

### 4.3. High-Performance Hybrid Fiber-Reinforced Concrete (HPHFRC)

In addition to the control mix (CM) of high-performance concrete, high-performance fiber-reinforced concrete (HPFRC) and high-performance hybrid fiber-reinforced concrete (HPHFRC) mixes were also cast using the same mix design. Kevlar fibers (KF) and glass fibers (GF) with lengths of 35 and 25 mm, respectively, were added and hybridized at 1.5% by mass of cement to create HPFRC and HPHFRC (GF: KF of 1.5%: 0%, 1.25%: 0.25%, 1%: 0.5%, 0.75%: 0.75%, 0.5%: 1%, 0.25%: 1.25%, 0%: 1.5%). The KF length was selected to counter macro-cracks and the GF length was selected to counter micro-cracks. Eight mixes were cast in total, including one CM, two HPFRC (M-G_1__.5_ and M-K_1__.5_), and five HPHFRC mixes (HF-G_1.25_K_0.25_, HF-G_1_K_0__.5_, HF-G_0__.75_K_0__.75_, HF-G_0.5_K_1_, and HF-G_0.25_K_1.25_). The “K” in the mix names represents Kevlar, “G” represents glass, and “HF” represents hybrid fibers. The subscript numbers in the mix names represent the percentages of fibers added by mass of cement, e.g., HF-G_0__.5_K_1_ is a hybrid fiber mix with 0.5% glass fibers by mass of cement and 1% Kevlar fibers by mass of cement. The fiber combinations of all mixes are given in Table 4.

### 4.4. Specimens and Testing

For every concrete mix, 9 standard cylinders (100 × 200 mm) were cast for every test, giving a total of 72 cylinders. Three of them were used for the compression test, three for the splitting tensile test, and the remaining three for the density and water absorption test. For each concrete mix, three standard beams (100 mm × 100 mm × 450 mm) were also cast to study the flexural behavior. The tests conducted in this study along with the ASTM codes are given in Table 5.

## 5. Test Results

### 5.1. Slump Behavior

Slump values of the control mix along with high-performance hybrid fiber mixes and other fiber mixes are shown in Table 6. Significant reductions in slump behavior were observed after the addition of fibers to the mixes. The slump behavior of M-K_1__.5_ was reduced by 32% as compared to M-G_1.5_. From M-G_1.5_ to M-K_1__.5_, the slump behavior was reduced due to the water absorption ability of the Kevlar fibers, while glass fibers on the other hand do not absorb water. The control mix was the most workable among the mixes. Special care was given to all fiber-reinforced and hybrid fiber-reinforced mixes when filling the molds.

### 5.2. Density and Water Absorption

The density values of the concrete mixes are shown in Table 7. The density levels of M-G_1__.5_, HF-G_1.25_K_0.25_, HF-G_1_K_0.5_, and HF-G_0.75_K_0.75_ increased by 7 kg/m^3^, 5.5 kg/m^3^, 2.5 kg/m^3^, and 1 kg/m^3^, respectively, as compared to CM. The increases in density were due to the addition of glass fibers, which have a greater density as compared to Kevlar fibers. The density levels of HF-G_0__.5_K_1_, HF-G_0.25_K_1.25_, and M-K_1.5_ decreased by 1.5 kg/m^3^, 4 kg/m^3^, and 5.5 kg/m^3^, respectively, as compared to CM. Decreases in density were observed as the percentage of Kevlar fibers increased in the hybrid mixes. This was due to the lower density of Kevlar fibers as compared to glass fibers. The water absorption levels of concrete mixes are shown in Table 7. It is noted that with increases in Kevlar fibers in the hybrid fiber mixes, the water absorption also increased. Kevlar fibers absorb more water than glass fibers. Furthermore, the density levels of Kevlar and glass fiber also significantly differ along with their other physical and mechanical properties.

### 5.3. Compressive Properties

**Figure 4:**
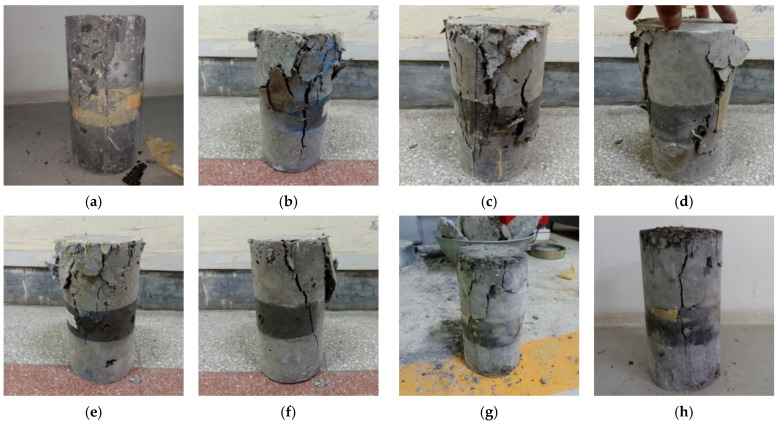
Compressive failure modes of specimens: (**a**) CM; (**b**) M-G_1__.5_; (**c**) HF-G_1.25_K_0.25_; (**d**) HF-G_1_K_0.5_; (**e**) HF-G_0.75_K_0.75_; (**f**) HF-G_0.5_K_1_; (**g**) HF-G_0.25_K_1.25_; (**h**) M-K_1.5_.

The 28 days compressive strength results for the concrete specimens are shown in Table 8. Increases in *f*′_c_ were noted for M-G_1__.5_, HF-G_1.25_K_0.25_, HF-G_1_K_0.5_, HF-G_0.75_K_0.75_, HF-G_0.5_K_1_, HF-G_0.25_K_1.25_, and M-K_1.5_ as compared to CM of 9.7%, 10.1%, 10.6%, 13.4%, 9.5%, 8.6%, and 9.4%, respectively. Significant increases in the compressive strength values of HPHFRC mixes were observed as compared to the CM and HPFRC mixes. This was due to the hybridization effect of the fibers. The failure modes under compressive loading for all specimens are shown in Figure 4.

**Figure 5:**
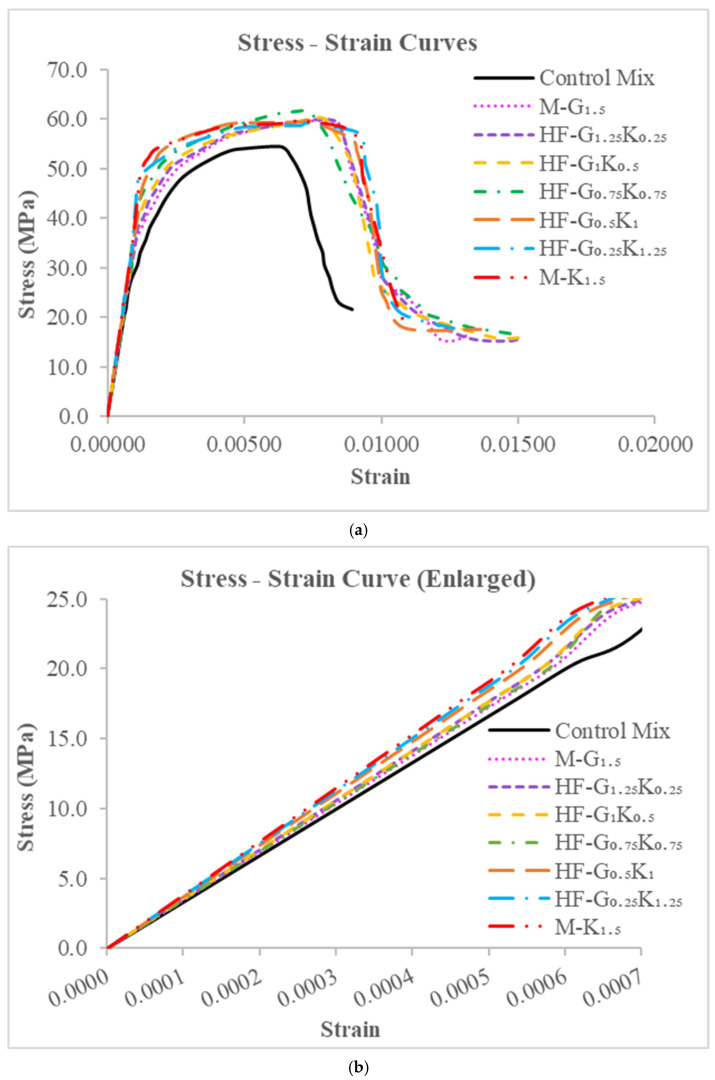
(**a**) Stress–strain curve under compressive loading and (**b**) enlarged stress–strain curves up to 25 MPa stress and 0.0007 strain.

The elastic modulus (Ec) values of all concrete mixes are given in Table 8. As compared to CM, the elastic modulus values of M-G_1__.5_, HF-G_1.25_K_0.25_, HF-G_1_K_0.5_, HF-G_0.75_K_0.75_, HF-G_0.5_K_1_, HF-G_0.25_K_1.25_, and M-K_1.5_ increased by 12.2%, 14.2%, 15.5%, 17.0%, 19.0%, 21.0%, and 22.6%, respectively. The elastic modulus of M-K_1__.5_ increased by 9.2% as compared to M-G_1.5_. The elastic modulus values were calculated from the stress–strain curves shown in Figure 5.

**Figure 6:**
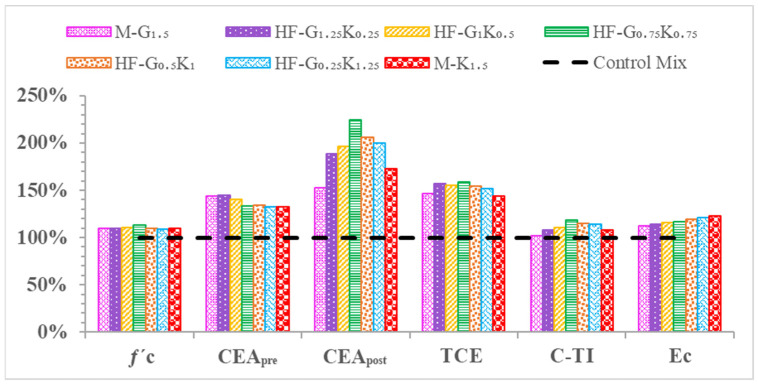
Percentage comparison of concrete properties during compression.

The energy absorbed before cracking, i.e., the compressive energy absorption pre-peak (CEA_pre_), is the area underneath the stress–strain curve from the beginning to peak stress. The energy absorbed after cracking, i.e., the compressive energy absorption post-peak (CEA_post_), is the area underneath the stress–strain curve from peak stress to failure stress. The summation of CEA_pre_ and CEA_post_ is regarded as the total compression energy absorption (TCE). The toughness index during compression (C-TI) is the ratio of the total compressive energy absorption to the compressive energy absorbed pre-peak (TCE/CEA_pre_). All of the described parameters under compression loading are shown in Table 8. The CEA_pre_ values of M-G_1__.5_, HF-G_1.25_K_0.25_, HF-G_1_K_0.5_, HF-G_0.75_K_0.75_, HF-G_0.5_K_1_, HF-G_0.25_K_1.25_, and M-K_1.5_ were increased by 43.9%, 44.5%, 40.2%, 33.5%, 34.4%, 32.8%, and 32.5%, respectively, as compared to CM. For CEA_post_, as compared to CM, increases of 52.4%, 88.2%, 96.0%, 124.6%, 105.8%, 100.3%, and 72.6% were observed for M-G_1__.5_, HF-G_1.25_K_0.25_, HF-G_1_K_0.5_, HF-G_0.75_K_0.75_, HF-G_0.5_K_1_, HF-G_0.25_K_1.25_, and M-K_1.5_, respectively. The highest CEA_post_ value was observed for HF-G_0__.75_K_0.75_, which may have been due to the hybridization effect of the fibers. The TCE values of M-G_1__.5_, HF-G_1.25_K_0.25_, HF-G_1_K_0.5_, HF-G_0.75_K_0.75_, HF-G_0.5_K_1_, HF-G_0.25_K_1.25_, and M-K_1.5_ were increased by 46.3%, 56.6%, 55.6%, 58.6%, 54.1%, 51.4%, and 43.6%, respectively, as compared to CM. The highest TCE value was observed for HF-G_0__.75_K_0.75_. It is noted that the C-TI values of M-G_1__.5_, HF-G_1.25_K_0.25_, HF-G_1_K_0.5_, HF-G_0.75_K_0.75_, HF-G_0.5_K_1_, HF-G_0.25_K_1.25_, and M-K_1.5_ increased by 1.6%, 8.3%, 11.0%, 18.8%, 14.6%, 14.0%, and 8.3%, respectively, as compared to CM. The percentages for *f*′_c_, CEApre, CEApost, TCE, C-TI, and E_c_ are given in Figure 6.

### 5.4. Flexural Properties

**Figure 7:**
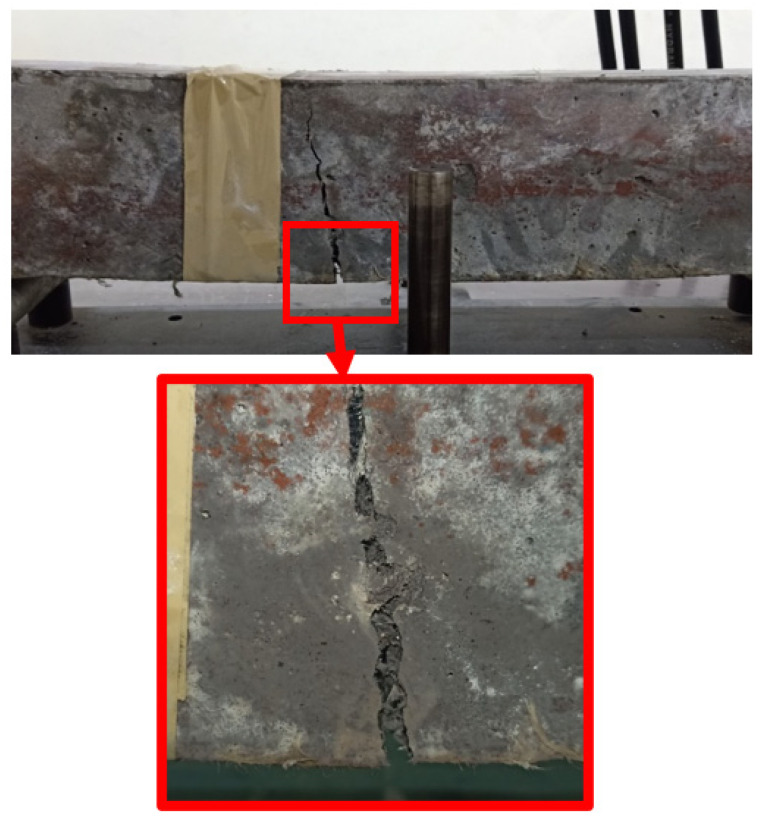
Bridging effects in HPFRC and HPHFRC specimens under flexural loading.

**Figure 8:**
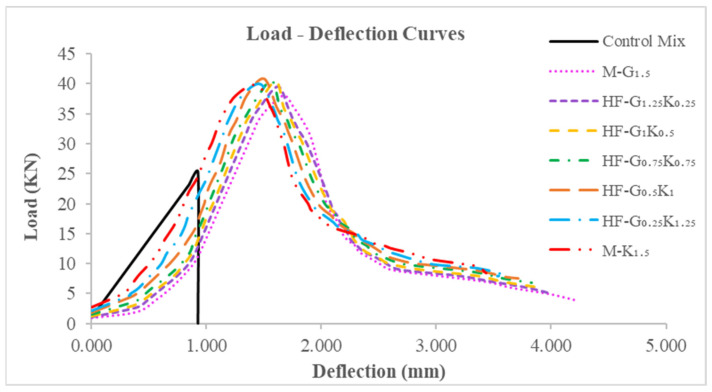
Load–deflection curves of concrete specimens.

The flexural strength (ƒᵣ) values of the beam specimens tested on the 28th day of curing are shown in Table 9. The flexural strength values of M-G_1__.5_, HF-G_1.25_K_0.25_, HF-G_1_K_0.5_, HF-G_0.75_K_0.75_, HF-G_0.5_K_1_, HF-G_0.25_K_1.25_ and M-K_1.5_ increased by 50.8%, 56.7%, 60.3%, 61.1%, 61.9%, 58.7%, and 57.9%, respectively, as compared to that of CM. The highest flexural strength was observed for the HF-G_0__.5_K_1_ specimen, which was the result of the hybridization of the fibers. The effect of loading under flexural loading is shown in Figure 7. The load deflection curves of concrete specimens under flexural loading are shown in Figure 8. The energy absorbed before cracking, i.e., the flexural energy absorbed pre-peak (FEApre), is the area underneath the load–deflection curve from the beginning to the peak load. The energy absorbed after cracking, i.e., the flexural energy absorption post-peak (FEApost), is the area underneath the load–deflection curve from peak load to failure load. The summation of FEA_pre_ and FEA_post_ is regarded as the total flexural energy absorption (TCE). The toughness index during flexural loading (F-TI) is ratio of the total flexural energy absorption to flexural energy absorbed pre-peak (TFE/FEA_pre_). All described parameters under compression loading are shown in Table 9. Bridging effects under flexural loading are shown in Figure 7. Increases of 123.7%, 125.3%, 131.7%, 134.9%, 132.1%, 145.4% and 162.3% were observed for FEA_pre_ values of M-G_1__.5_, HF-G_1.25_K_0.25_, HF-G_1_K_0.5_, HF-G_0.75_K_0.75_, HF-G_0.5_K_1_, HF-G_0.25_K_1.25_, and M-K_1.5_, respectively. The highest FEA_pre_ was observed for M-K_1__.5_. The FEA_post_ for CM was observed to be zero because it failed after the first crack and could not absorb energy after the maximum load. Therefore, the increases in the FEA_post_ values of HF-G_1__.25_K_0.25_, HF-G_1_K_0.5_, HF-G_0.75_K_0.75_, HF-G_0.5_K_1_, HF- G_0__.25_K_1.25_, and M-K_1.5_ specimens equaled 4.6%, 6.5%, 9.0%, 13.4%, 12.7%, and 8.3%, respectively, as compared to M-G_1.5_, which was increased by 100% as compared to CM.

**Figure 9:**
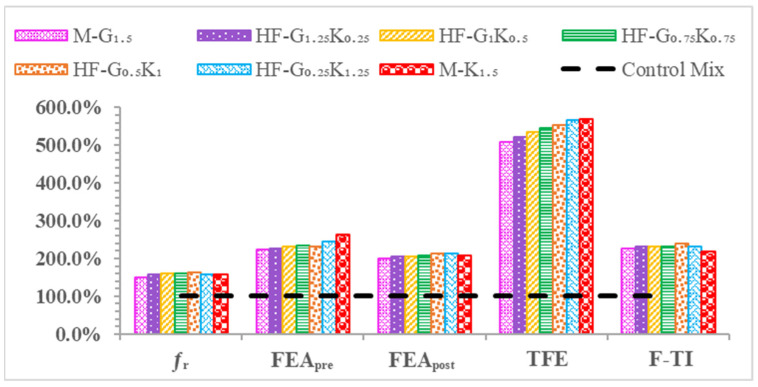
Percentage comparison of concrete properties under flexural loading.

The highest FEA_post_ value was noted for HF-G_0__.5_K_1_. The increases in TEF as compared to CM were 407.9%, 422.5%, 434.2%, 444.7%, 454.3%, 465.6%, and 470.1% for concrete specimens of M-G_1__.5_, HF-G_1.25_K_0.25_, HF-G_1_K_0.5_, HF-G_0.75_K_0.75_, HF-G_0.5_K_1_, HF-G_0.25_K_1.25_, and M-K_1.5_, respectively. The highest TEF value was noted for M-K_1__.5_. For F-TI values, increases of 127.0%, 131.9%, 130.6%, 131.9%, 138.8%, 130.4%, and 117.3% were observed for concrete specimens of M-G_1.5_, HF-G_1.25_K_0.25_, HF-G_1_K_0.5_, HF-G_0.75_K_0.75_, HF-G_0.5_K_1_, HF-G_0.25_K_1.25_, and M-K_1.5_, respectively, as compared to CM. The highest F-TI value was observed for HF-G_0__.5_K_1_. A percentage comparison of the concrete properties under the influence of flexural loading is shown in Figure 9.

### 5.5. Splitting Tensile Properties

**Figure 10:**
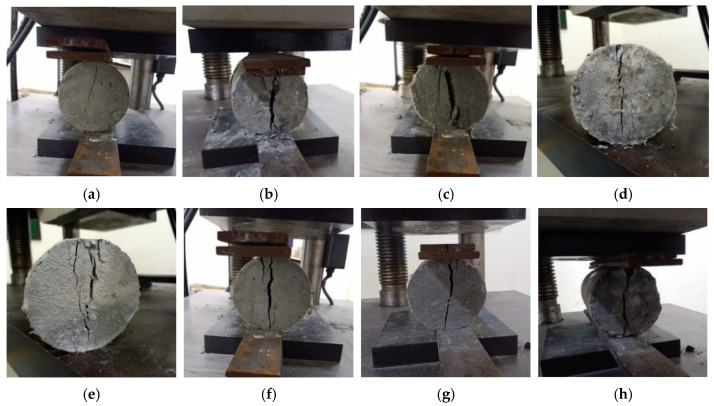
Splitting tensile failure modes of specimens: (**a**) CM; (**b**) M-G_1__.5_; (**c**) HF-G_1.25_K_0.25_; (**d**) HF-G_1_K_0.5_; (**e**) HF-G_0.75_K_0.75_; (**f**) HF-G_0.5_K_1_; (**g**) HF-G_0.25_K_1.25_; (**h**) M-K_1__.5_.

The splitting tensile strength (*f*_s_) values of cylindrical specimens are shown in Table 10. The increases in *f*_s_ for M-G_1__.5_, HF-G_1.25_K_0.25_, HF-G_1_K_0.5_, HF-G_0.75_K_0.75_, HF-G_0.5_K_1_, HF-G_0.25_K_1.25_, and M-K_1.5_ as compared to CM were observed to be 26.5%, 27.5%, 29.3%, 27.8%, 28.5%, 27.3%, and 25.9%, respectively. The highest *f*_s_ was observed for the HF-G_1_K_0__.5_ specimen, which was a hybrid fiber mix. The failure modes under splitting tensile loading for all specimens are shown in Figure 10.

**Figure 11:**
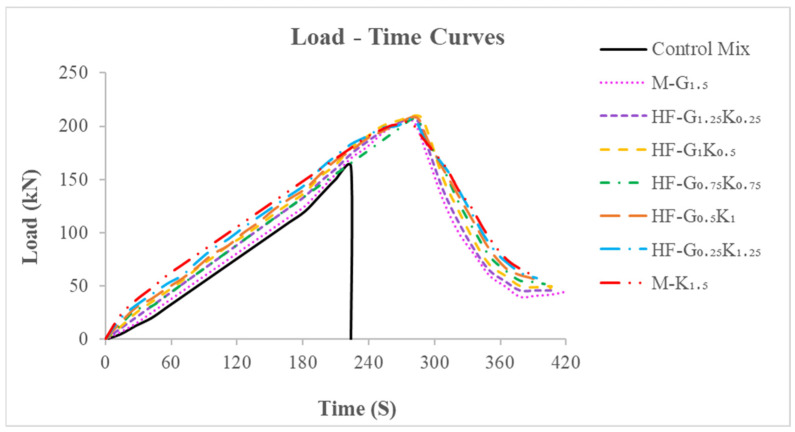
Load–time curves of concrete specimens.

**Figure 12:**
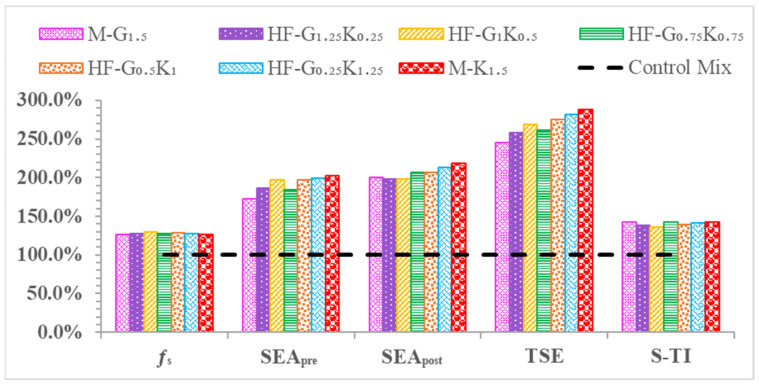
Percentage comparison of concrete properties under splitting tensile loading.

The splitting tensile energy absorption pre-peak (SEA_pre_) is the area underneath the load–time curve from the beginning to the peak load. The splitting tensile energy absorption post-peak (SEA_post_) is the area underneath the load–time curve from the peak load to the failure load. The summation of the SEA_pre_ and SEA_post_ values is regarded as the total splitting tensile energy absorption (TSE). The splitting tensile failure modes of the specimens are shown in Figure 10, while the load-time curves of the specimens can be seen in Figure 11. The toughness index during splitting tensile loading (S-TI) is the ratio of the total splitting tensile energy absorption to splitting tensile energy absorption pre-peak (TSE/SEA_pre_). The SEA_pre_, SEA_post_, TSE, and S-TI values for the concrete specimens are given in Table 10. The increases in SEA_pre_ for M-G_1__.5_, HF-G_1.25_K_0.25_, HF-G_1_K_0.5_, HF-G_0.75_K_0.75_, HF-G_0.5_K_1_, HF-G_0.25_K_1.25_, and M-K_1.5_ as compared to CM were observed to be 72.3%, 86.4%, 97.4%, 84.1%, 97.4%, 99.6%, and 101.9%, respectively. The highest SEA_pre_ was noted for M-K_1__.5_. The decreases in SEA_post_ for HF-G_1__.25_K_0.25_ and HF-G_1_K_0.5_ equaled 1.5% and 1.8%, respectively, as compared to M-G_1.5_. For SEA_post_, the increases for HF-G_0__.75_K_0.75_, HF-G_0.5_K_1_, HF-G_0.25_K_1.25_, and M-K_1.5_ equaled 6.4%, 6.5%, 12.9%, and 19.0%, respectively. The highest value was observed for M-K_1__.5_. The increases in TSE for M-G_1.5_, HF-G_1.25_K_0.25_, HF-G_1_K_0.5_, HF-G_0.75_K_0.75_, HF-G_0.5_K_1_, HF-G_0.25_K_1.25_, and M-K_1.5_ as compared to CM equaled 144.9%, 157.9%, 168.7%, 161.3%, 174.7%, 181.6%, and 188.4%, respectively. The increases in S-TI for M-G_1__.5_, HF-G_1.25_K_0.25_, HF-G_1_K_0.5_, HF-G_0.75_K_0.75_, HF-G_0.5_K_1_, HF-G_0.25_K_1.25_, and M-K_1.5_ as compared to CM equaled 42.2%, 38.4%, 36.1%, 42.0%, 39.2%, 41.1%, and 42.8%, respectively. The highest S-TI was noted for M-K_1__.5_. A percentage comparison of the concrete specimens under splitting tensile loading is given in Figure 12.

## 6. Discussion

**Figure 13:**
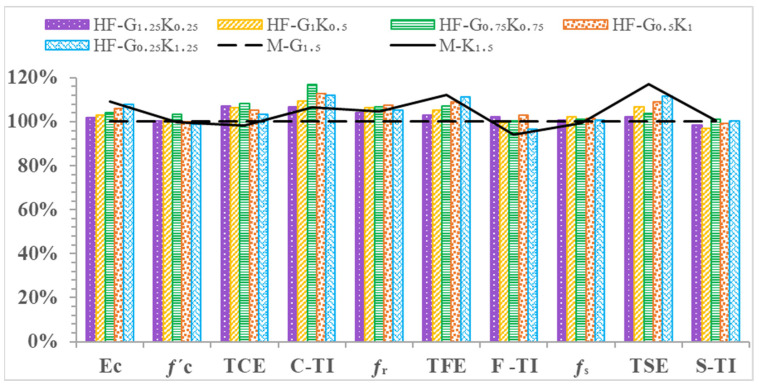
Comparison of the properties of HPHFRC and HPFRC.

The optimization of concrete mixes is shown in Table 11. The mix with the best results was HF-G_0__.75_K_0.75_. The HF-G_0__.75_K_0.75_ mix is recommended for rehabilitation purposes. The E_c_, *f*′_c_, TCE, C-TI, *f*_r_, TFE, F-TI, *f*_s_, TSE, and S-TI values of recommended mix (HF-G_0__.75_K_0.75_) increased by 14.6%, 11.8%, 35.0%, 15.9%, 37.9%, 81.6%, 56.9%, 21.7%, 61.7%, and 29.6%, respectively, as compared to CM. The results for HF-G_0__.75_K_0.75_ were the best, as shown in Table 11. The top three mixes with the most enhanced properties were hybrid fiber mixes (HF-G_1_K_0__.5_, HF-G_0__.75_K_0.75_, and HF-G_0.5_K_1_). These mixes can be used for rehabilitation work. Based on the above results, hybrid fiber concrete can be used in rehabilitation because of its enhanced mechanical properties in comparison to fiber-reinforced concrete. A comparison of the properties of HFRC with FRC is given in Figure 13.

## 7. Empirical Equations for Predicting Toughness Index

**Figure 14:**
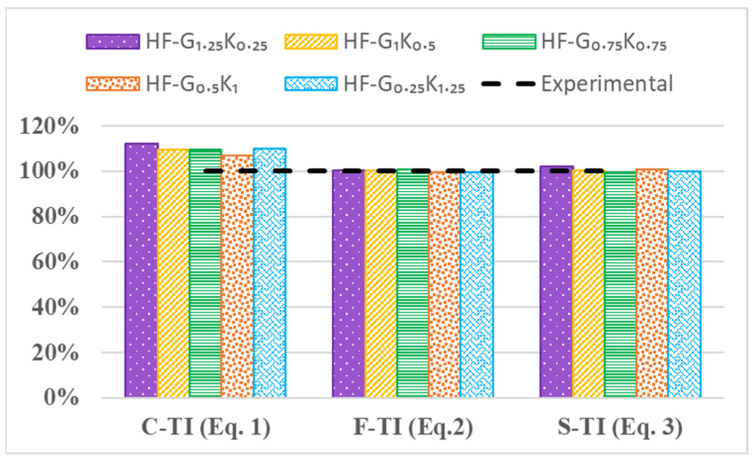
Comparison of the HPHFRC’s experimental and empirical toughness index values.

The toughness index shows the energy absorption of a material post-fracture, which is given primary importance when choosing a material for rehabilitation. The empirical equations (Equations (1)–(3)) for predicting toughness indexes (C-TI, F-TI, and S-TI) were developed for fiber percentages by using experimental data from compressive, flexural, and splitting tensile strength tests. The *R*^2^ values ranged from 0.66 to 0.84 and the maximum variance observed between experimental and numerical values was 12%, as shown in Figure 14.

For the compression toughness index:

(1) $$\begin{array}{cc} C-TI=0.0494{\left({f^{\prime }}_{c}\right)}^{2}- & 5.9515\left({f^{\prime }}_{c}\right)+180.93\\ & \left(R^{2}=0.758\right) \end{array}$$

For the flexural toughness index:

(2) $$\begin{array}{cc} F-TI=0.6619{\left(f_{r}\right)}^{2}- & 23.832\left(f_{r}\right)+216.81\\ & \left(R^{2}=0.8383\right) \end{array}$$

For the splitting tensile toughness index:

(3) $$\begin{array}{cc} S-TI=-7.1472{\left(f_{s}\right)}^{2}+ & 94.237\left(f_{s}\right)-309.22\\ & \left(R^{2}=0.6621\right) \end{array}$$

## 8. Conclusions

Significant enhancements in the overall properties of high-performance hybrid fiber-reinforced concrete (HPHFRC) were observed as compared to high-performance fiber-reinforced concrete (HPFRC) and the control mix (CM), proving the usefulness of the fiber hybridization method in the concrete industry. In the rehabilitation of concrete bridges and structures, these enhancements can be put to use.

The following are some of the conclusions extracted from this study:

1. The workability of concrete was significantly reduced with the addition of fibers. As the percentage of Kevlar fibers increased in the concrete, the workability decreased due to the water absorption of Kevlar fibers;
2. The maximum density was observed for mixes with dominant glass fiber percentages (M-G_1__.5_, HF-G_1.25_K_0.25_, HF-G_1_K_0.5_, HF-G_0.75_K_0.75_), because glass fibers are denser than Kevlar fiber. A correlation with the minimum water absorption was also observed in glass-fiber-dominant mixes. This was due to the water absorption properties of the Kevlar fibers;
3. The overall best properties were observed for the hybrid mix (HF-G_0__.75_K_0.75_) with 0.75% of glass fibers and 0.75% of Kevlar fibers. Among all mixes, the most dominant properties were for the HF-G_0__.75_K_0.75_ mix due to the equal quantities of fibers exhibiting the best synergetic effect;
4. The elastic modulus of HF-G_0__.75_K_0.75_ increased by 17% as compared to CM, which was very near to the maximum value observed. The compressive strength, total compressive energy, and toughness index during compression for HF-G_0__.75_K_0.75_ were increased by 13.4%, 58.6%, and 18.8% as compared to CM, which were the maximum values observed out of all mixes;
5. The flexural strength, total flexural energy absorbed, and toughness index during flexural loading for HF-G_0__.75_K_0.75_ increased by 61.1%, 444.7%, and 131.9% as compared to CM;
6. As compared to CM, increases of 127.8%, 261.3%, and 142% were observed in terms of the splitting tensile strength, total splitting tensile energy absorbed, and splitting tensile toughness index of HF-G_0__.75_K_0.75_;
7. The high-performance hybrid fiber-reinforced concrete illustrated the best performance as compared to the CM and HPFRC mixes. The top three mixes that showed the best properties were hybrid fiber-reinforced concrete mixes (HF-G_1_K_0__.5_, HF-G_0.75_K_0.75_, HF-G_0__.5_K_1_);
8. Based on these conclusions, the hybrid fiber mixes demonstrated more enhanced overall properties as compared to FRCs. These improved mechanical properties of the HFRC can be utilized in the rehabilitation of bridges and structures.

## 9. Recommendations

A long-term durability study of HPHFRC for rehabilitation needs to be performed.

The chemical resistance should be evaluated before the practical implementation of the HFRC.

Fibers of different length should be used to check their hybridization effects.

To improve the mix’s workability, different super plasticizers need to be checked to identify the best results.

A cost analysis is recommended before any commercial application.

## Figures and Tables

**Table 2:** Mix proportions of the control mix.

Materials	Weight (kg/m^3^)
Ordinary Portland cement	500
Fine sand	600
Silica fumes	40
Coarse aggregate (granite crush ≈ 10 mm)	594
Coarse aggregate (Margala crush ≈ 5 mm)	306
Water (W/C + SF = 0.31)	167
High-performance water-reducing admixture (SP-303)	3 L

**Table 3:** Physical and mechanical properties of fibers (*DuPont™ Kevlar^®^ 29 Aramid Fiber*, n.d.; *E-Glass Fiber*, *Generic*, n.d.).

Properties	Fiber Type	
	Kevlar Fibers	Glass Fibers
Tensile Strength (MPa)	3620	3450
Elongation at Break	3.6%	4.8%
Modulus of Elasticity (GPa)	70.3	72.4
Fiber Length (mm)	35	25
Sectional Dimension (μm)	23	14
Density (g/cc)	1.44	2.60

**Table 4:** Fiber combinations by percentage weight of cement.

Mix Type	Percentage of Fiber by Weight of Cement (kg/m^3^)	
	Glass Fibers (GF)	Kevlar Fibers (KF)
CM	0	0
M-G_1__.5_	1.5 (7.5)	0
HF-G_1__.25_K_0.25_	1.25 (6.25)	0.25 (1.25)
HF-G_1_K_0__.5_	1 (5)	0.5 (2.5)
HF-G_0__.75_K_0.75_	0.75 (3.75)	0.75 (3.75)
HF-G_0__.5_K_1_	0.5 (2.5)	1 (5)
HF-G_0__.25_K_1.25_	0.25 (1.25)	1.25 (6.25)
M-K_1__.5_	0	1.5 (7.5)

**Notes**: Data in parentheses show kg/m^3^ values of fibers in mix.

**Table 5:** Tests conducted and parameters measured in this study.

Tests	ASTM Standards	Parameters Measured
Slump	ASTM C143 [37]	Workability of mixes
Density and Water absorption	ASTM C642 [38]	Density of mix in kg/m^3^ and water absorption as a percentage
Compressive strength	ASTM C39, ASTM C469 [39]	Compressive strength (*f*′_c_), elastic modulus (E_c_), Compression energy absorbed pre-peak (CEA_pre_), Compression energy absorbed post-peak (CEA_post_), total compression energy absorbed (TCE), compression toughness index (C-TI)
Splitting tensile strength	ASTM C496 [40]	Splitting tensile strength (*f*_s_), splitting tensile energy absorbed pre-peak (SEA_pre_), splitting tensile energy absorbed post-peak (SEA_post_), total splitting tensile energy absorbed (TSE), splitting tensile toughness index (S-TI)
Flexural strength	ASTM C1609 [41]	Flexural strength (*f*_r_), flexural energy absorbed pre-peak (FEA_pre_), flexural energy absorbed post-peak (FEA_post_), total flexural energy absorbed (TFE), flexural toughness index (F-TI)

**Table 6:** Slump behavior of CM and hybrid fiber mixes.

Concrete Type	Slump (mm)	Slump Reduction Compared to CM %
Control Mix (CM)	70	(-)
M-G_1__.5_	50	29%
HF-G_1__.25_K_0.25_	47	33%
HF-G_1_K_0__.5_	45	36%
HF-G_0__.75_K_0.75_	44	37%
HF-G_0__.5_K_1_	41	41%
HF-G_0__.25_K_1.25_	37	47%
M-K_1__.5_	34	51%

**Table 7:** Density and water absorption levels of concrete mixes.

Concrete Type	Density (kg/m^3^)	Water Absorption (%)
CM	2567	1.73
M-G_1__.5_	2574	1.46
HF-G_1__.25_K_0.25_	2572.5	1.57
HF-G_1_K_0__.5_	2569.5	1.63
HF-G_0__.75_K_0.75_	2568	1.70
HF-G_0__.5_K_1_	2565.5	1.79
HF-G_0__.25_K_1.25_	2563	1.84
M-K_1__.5_	2561.5	1.94

**Table 8:** Properties of concrete during compression.

Concrete Type	Parameters					
	*f*’ _c_	E_c_	CEA_pre_	CEA_post_	TCE	C-TI
	(MPa)	(GPa)	(MPa)	(MPa)	(MPa)	(-)
CM	54.5 ± 0.9	32.2 ± 1.5	0.274 ± 0.001	0.104 ± 0.001	0.378 ± 0.001	1.381 ± 0.04
M-G_1__.__5_	59.8 ± 0.8	36.1 ± 1.2	0.394 ± 0.002	0.159 ± 0.001	0.553 ± 0.002	1.404 ± 0.05
HF-G_1__.__25_K_0__.__25_	60.0 ± 1.2	36.8 ± 1.2	0.396 ± 0.001	0.197 ± 0.001	0.592 ± 0.001	1.496 ± 0.03
HF-G_1_K_0__.__5_	60.3 ± 1.0	37.2 ± 1.3	0.384 ± 0.001	0.205 ± 0.001	0.589 ± 0.002	1.533 ± 0.04
HF-G_0__.__75_K_0__.__75_	61.8 ± 0.7	37.7 ± 0.8	0.366 ± 0.002	0.234 ± 0.001	0.600 ± 0.002	1.641 ± 0.02
HF-G_0__.__5_K_1_	59.7 ± 0.9	38.3 ± 1.1	0.368 ± 0.001	0.215 ± 0.001	0.583 ± 0.001	1.583 ± 0.01
HF-G_0__.__25_K_1__.__25_	59.2 ± 0.8	39.0 ± 0.9	0.364 ± 0.002	0.209 ± 0.001	0.573 ± 0.002	1.575 ± 0.03
M-K_1__.__5_	59.6 ± 1.1	39.5 ± 0.9	0.363 ± 0.001	0.180 ± 0.001	0.543 ± 0.001	1.496 ± 0.02

**Table 9:** Properties of concrete specimens under flexural loading.

Concrete Type	Parameters				
	*f*_r_	FEA_pre_	FEA_post_	TFE	F-TI
	(MPa)	(J)	(J)	(J)	(-)
CM	11.34 ± 0.3	10.53 ± 0.8	0.00 ± 0.0	10.53 ± 0.8	1.00 ± 0.0
M-G_1__.__5_	17.10 ± 0.5	23.57 ± 0.9	29.93 ± 0.9	53.50 ± 1.0	2.27 ± 0.01
HF-G_1__.__25_K_0__.__25_	17.78 ± 0.5	23.73 ± 0.6	31.31 ± 0.8	55.04 ± 1.1	2.32 ± 0.01
HF-G_1_K_0__.__5_	18.18 ± 0.2	24.40 ± 0.5	31.87 ± 1.0	56.28 ± 0.8	2.31 ± 0.01
HF-G_0__.__75_K_0__.__75_	18.27 ± 0.1	24.74 ± 0.6	32.63 ± 0.8	57.38 ± 0.9	2.32 ± 0.01
HF-G_0__.__5_K_1_	18.36 ± 0.3	24.45 ± 0.7	33.94 ± 0.9	58.39 ± 0.8	2.39 ± 0.01
HF-G_0__.__25_K_1__.__25_	18.00 ± 0.4	25.86 ± 0.4	33.72 ± 1.1	59.58 ± 1.2	2.30 ± 0.02
M-K_1__.__5_	17.91 ± 0.2	27.64 ± 0.4	32.42 ± 0.8	60.05 ± 0.7	2.17 ± 0.01

**Table 10:** Properties of concrete specimens under splitting tensile loading.

Concrete Type	Parameters				
	*f*_s_	SEA_pre_	SEA_post_	TSE	S-TI
	(MPa)	(kN.s)	(kN.s)	(kN.s)	(-)
CM	5.16 ± 0.6	16,156 ± 100	0 ± 0.0	16,156 ± 100	1.00 ± 0.0
M-G_1__.__5_	6.53 ± 0.8	28,993 ± 120	11,732 ± 150	39,565 ± 130	1.40 ± 0.03
HF-G_1__.__25_K_0__.__25_	6.58 ± 0.7	30,112 ± 150	11,552 ± 190	41,664 ± 140	1.38 ± 0.05
HF-G_1_K_0__.__5_	6.67 ± 0.8	31,886 ± 180	11,526 ± 120	43,412 ± 150	1.36 ± 0.02
HF-G_0__.__75_K_0__.__75_	6.59 ± 0.7	29,739 ± 200	12,484 ± 145	42,223 ± 160	1.42 ± 0.03
HF-G_0__.__5_K_1_	6.63 ± 0.7	31,888 ± 220	12,500 ± 160	44,388 ± 180	1.39 ± 0.06
HF-G_0__.__25_K_1__.__25_	6.57 ± 0.6	32,251 ± 160	13,247 ± 170	45,497 ± 160	1.41 ± 0.02
M-K_1__.__5_	6.50 ± 0.7	33,706 ± 140	13,961 ± 160	46,587 ± 150	1.41 ± 0.02

**Table 11:** Comparison of minimum, maximum, and recommended mixes.

Parameters	Mix with Minimum Values	Mix with Maximum Values	Recommended Mix Values (HF-G_0__.__75_K_0__.__75_)
**E_c_** (GPa)	32.2 ± 1.5 (CM)	39.5 ± 0.9 (M-K_1__.__5_)	37.7 ± 1.5
***f*′_c_** (MPa)	54.5 ± 0.9 (CM)	61.8 ± 0.7 (HF-G_0__.__75_K_0__.__75_)	61.8 ± 0.7
**TCE** (MPa)	0.378 ± 0.001 (CM)	0.6 ± 0.002 (HF-G_0__.__75_K_0__.__75_)	0.6 ± 0.002
**C-TI** (-)	1.38 ± 0.04 (CM)	1.64 ± 0.02 (HF-G_0__.__75_K_0__.__75_)	1.64 ± 0.02
***f*_r_** (MPa)	11.34 ± 0.3 (CM)	18.36 ± 0.3 (HF-G_0__.__5_K_1_)	18.27 ± 0.3
**TFE** (J)	10.53 ± 0.8 (CM)	60.05 ± 0.7 (M-K_1__.__5_)	60.05 ± 0.7
**F-TI** (-)	1 ± 0.0 (CM)	2.39 ± 0.01 (HF-G_0__.__5_K_1_)	2.32 ± 0.01
***f*_s_** (MPa)	5.16 ± 0.6 (CM)	6.67 ± 0.8 (HF-G_1_K_0__.__5_)	6.67 ± 0.8
**TSE** (kN.s)	16,156 ± 100 (CM)	46,587 ± 150 (M-K_1__.__5_)	42,587 ± 150
**S-TI** (-)	1 ± 0.0 (CM)	1.42 ± 0.03 (HF-G_0__.__75_K_0__.__75_)	1.42 ± 0.03

## Data Availability

The data can be made available from the corresponding author upon reasonable request.

## References

[1] Hossain M.A., Rahman M., Morshed A.Z., Haque S.M. Investigation of The Effect of Nylon Fiber in Concrete Rehabilitation. International Conference on Civil Engineering for Sustainable Development. https://www.researchgate.net/publication/326646258_Investigation_of_the_Effect_of_Nylon_Fiber_in_Concrete_Rehabilitation.

[2] Banthia N., Zanotti C., Sappakittipakorn M. (2014). Sustainable fiber reinforced concrete for repair applications. Constr. Build. Mater..

[3] Nguyen-Minh L., RovňÁk M., Tran-Quoc T., Nguyen-Kim K. (2011). Punching shear resistance of steel fiber reinforced concrete flat slabs. Procedia Eng..

[4] Plizzari G.A. (2018). Fiber Reinforced Concrete for repairing and strengthening RC structures: Some recent advancements. MATEC Web Conf..

[5] Michels J., Waldmann D., Maas S., Zürbes A. (2012). Steel fibers as only reinforcement for flat slab construction—Experimental investigation and design. Constr. Build. Mater..

[6] Natural vs. Synthetic Fiber Reinforced Polymer. Retrieved 25 August 2020. https://www.ukessays.com/essays/construction/natural-vs-synthetic-fiber-reinforced-polymer-construction-essay.php?vref=1.

[7] Chasioti S.G., Vecchio F.J. (2017). Effect of fiber hybridization on basic mechanical properties of concrete. ACI Struct. J..

[8] Tayeh B.A., Naja M.A., Shihada S., Arafa M. (2019). Repairing and strengthening of damaged RC columns using thin concrete jacketing. Adv. Civ. Eng..

[9] Brühwiler E., Denarié E. (2013). Rehabilitation and strengthening of concrete structures using ultra-high performance fibre reinforced concrete. Struct. Eng. Int. J. Int. Assoc. Bridge Struct. Eng. (IABSE).

[10] Kene K.S. (2012). Experimental Study on Behavior of Steel and Glass Fiber Reinforced Concrete Composites. Bonfring Int. J. Ind. Eng. Manag. Sci..

[11] Júlio E.S., Branco F., Silva V.D. (2003). Structural rehabilitation of columns with reinforced concrete jacketing. Prog. Struct. Eng. Mater..

[12] Algburi A.H.M., Sheikh M.N., Hadi M.N.S. (2019). New technique for strengthening square-reinforced concrete columns by the circularisation with reactive powder concrete and wrapping with fibre-reinforced polymer. Struct. Infrastruct. Eng..

[13] Chalioris C.E., Pourzitidis C.N. (2012). Rehabilitation of Shear-Damaged Reinforced Concrete Beams Using Self-Compacting Concrete Jacketing. ISRN Civ. Eng..

[14] Brühwiler E. (2020). UHPFRC technology to enhance the performance of existing concrete bridges. Struct. Infrastruct. Eng..

[15] Chen H.J., Yu Y.L., Tang C.W. (2020). Mechanical properties of ultra-high performance concrete before and after exposure to high temperatures. Materials.

[16] Smarzewski P. (2019). Processes of cracking and crushing in hybrid fibre reinforced high-performance concrete slabs. Processes.

[17] Al-Gemeel A.N., Zhuge Y., Youssf O. (2018). Use of hollow glass microspheres and hybrid fibres to improve the mechanical properties of engineered cementitious composite. Constr. Build. Mater..

[18] Khan M., Cao M., Ali M. (2018). Effect of basalt fibers on mechanical properties of calcium carbonate whisker-steel fiber reinforced concrete. Constr. Build. Mater..

[19] Dawood E.T., Ramli M. (2018). Production of Durable High Strength Flowable Mortar Reinforced with Hybrid Fibers. Chall. J. Concr. Res. Lett..

[20] Afroughsabet V., Ozbakkaloglu T. (2015). Mechanical and durability properties of high-strength concrete containing steel and polypropylene fibers. Constr. Build. Mater..

[21] Almusallam T.H., Abadel A.A., Al-Salloum Y.A., Siddiqui N.A., Abbas H. (2015). Effectiveness of hybrid-fibers in improving the impact resistance of RC slabs. Int. J. Impact Eng..

[22] Chi Y., Xu L., Zhang Y. (2014). Experimental study on hybrid fiber-reinforced concrete subjected to uniaxial compression. J. Mater. Civ. Eng..

[23] Soe K.T., Zhang Y.X., Zhang L.C. (2013). Material properties of a new hybrid fibre-reinforced engineered cementitious composite. Constr. Build. Mater..

[24] Bajaj V., Singh A.P., Singh S.P., Kaushik S.K. (2012). Flexural fatigue analysis of hybrid fibre-reinforced concrete. Mag. Concr. Res..

[25] Dawood E.T., Ramli M. (2011). Contribution of hybrid fibers on the hybrid fibers on the properties of high strength concrete having high workability. Procedia Eng..

[26] Banthia N., Sappakittipakorn M. (2007). Toughness enhancement in steel fiber reinforced concrete through fiber hybridization. Cem. Concr. Res..

[27] Sivakumar A., Santhanam M. (2007). Mechanical properties of high strength concrete reinforced with metallic and non-metallic fibres. Cem. Concr. Compos..

[28] Ahmed SF U., Maalej M., Paramasivam P. (2007). Flexural responses of hybrid steel-polyethylene fiber reinforced cement composites containing high volume fly ash. Constr. Build. Mater..

[29] Hua Y., Lian J.-Y., Zhou T.-Q. Relationship between the Mechanical Properties of Hybrid Fiber Reinforced Concrete and Length/Diameter Aspect Ratio of Hybrid Fiber. https://www.researchgate.net/publication/292543754_Relationship_between_the_mechanical_properties_of_hybrid_fiber_reinforced_concrete_and_lengthdiameter_aspect_ratio_of_hybrid_fiber.

[30] Lawler J.S., Zampini D., Shah S.P. (2005). Microfiber and macrofiber hybrid fiber-reinforced concrete. J. Mater. Civ. Eng..

[31] Yao W., Li J., Wu K. (2003). Mechanical properties of hybrid fiber-reinforced concrete at low fiber volume fraction. Cem. Concr. Res..

[32] Lawler J.S., Zampini D., Shah S.P. (2002). Permeability of cracked hybrid fiber-reinforced mortar under load. ACI Mater. J..

[33] Ramanalinagm N., Paramasivam P., Mansur M.A., Maalej M.M. Flexural Behavior of Hybrid Fiber Reinforced Cement Composites Containing High Volume Fly Ash|Request PDF. Proceedings of the 7th CANMET/ACI International Conference on Fly Ash, Silica Fume, Slag and Natural Pozzolans in Concrete.

[34] Sun W., Chen H., Luo X., Qian H. (2001). The effect of hybrid fibers and expansive agent on the shrinkage and permeability of high-performance concrete. Cem. Concr. Res..

[35] Cao J., Liu L., Zhao S. (2020). Relationship between Corrosion of Reinforcement and Surface Cracking Width in Concrete. Adv. Civ. Eng..

[36] Shi X. (2018). Monitoring of reinforced concrete corrosion. Eco-Efficient Repair and Rehabilitation of Concrete Infrastructures.

[37] (2018). Standard Test Method for Slump of Hydraulic-Cement Concrete.

[38] (2013). Standard Test Method for Density, Absorption, and Voids in Hardened Concrete.

[39] (2008). Standard Test Method for Compressive Strength of Cylindrical Concrete Specimens.

[40] (2004). Standard Test Method for Splitting Tensile Strength of Cylindrical Concrete.

[41] (2006). Standard Test Method for Flexural Performance of Fiber Reinforced Concrete (Using Beam with Third-Point Loading).

